# In Silico Network Pharmacology, Molecular Docking, and Molecular Dynamics Analysis of Rosemary-Derived Compounds as Potential HSP90 Inhibitors for Cancer Therapy

**DOI:** 10.3390/cimb47100860

**Published:** 2025-10-18

**Authors:** Radhia Mazri, Mebarka Ouassaf, Afaf Zekri, Shafi Ullah Khan, Kannan R. R. Rengasamy, Bader Y. Alhatlani

**Affiliations:** 1 Group of Computational and Medicinal Chemistry, LMCE Laboratory, University of Biskra, Biskra 07000, Algeria; radhia.mazri@univ-biskra.dz (R.M.); afaf.zekri@univ-biskra.dz (A.Z.); 2 Inserm U1086 ANTICIPE (Interdisciplinary Research Unit for Cancer Prevention and Treatment), Normandie Univ, Université de Caen Normandie, 14076 Caen, France; shafiullahpharmd@gmail.com; 3 Comprehensive Cancer Center François Baclesse, UNICANCER, 14076 Caen, France; 4 Laboratory of Natural Products and Medicinal Chemistry (LNPMC), Department of Pharmacology, Saveetha Dental College and Hospitals, Saveetha Institute of Medical and Technical Sciences (SIMATS), Thandalam, Chennai 602105, India; kannan@lnpmc.in; 5 Centre of Excellence for Pharmaceutical Sciences, North-West University, Potchefstroom 2520, South Africa; 6 Unit of Scientific Research, Applied College, Qassim University, Buraydah 52571, Saudi Arabia

**Keywords:** rosemary, cancer, protein–protein interaction, molecular docking, HSP90, molecular dynamics, ADMET

## Abstract

Cancer remains a major global health challenge, emphasizing the need for new and effective therapies. This study investigates the anticancer potential of bioactive compounds from rosemary (Rosmarinus officinalis) using an integrative network pharmacology and computational approach. Twelve phytochemicals with favorable pharmacological profiles, optimal pharmacokinetics, and acceptable toxicological properties were evaluated, revealing 178 putative cancer-related targets. Protein–protein interaction (PPI) analysis highlighted ten key genes—EGFR, ESR1, HIF1A, HSP90AA1, MAPK1, BCL2, STAT3, TP53, CASP3, and SRC—implicated in the progression of various cancers, including breast, colorectal, liver, and lung tumors. Functional enrichment analysis demonstrated their involvement in multiple cancer-associated pathways. Among these, HSP90AA1 emerged as a critical target. Molecular docking revealed Rosmanol, Chlorogenic acid, and Carnosol as the most promising HSP90AA1 binders with strong predicted affinities. ADMET profiling confirmed their excellent drug-likeness and safety profiles, while molecular dynamics simulations validated the stability of the compound–protein complexes, further supporting their potential as HSP90 inhibitors. These findings suggest that rosemary-derived compounds may represent valuable candidates for anticancer drug development, though experimental validation is required to confirm their therapeutic efficacy.

## 1. Introduction

Cancer remains one of the leading causes of mortality worldwide, characterized by uncontrolled cell proliferation and metastasis. Its complexity lies in its ability to evade normal regulatory mechanisms, leading to aberrant cell growth and the potential to invade other tissues. This phenomenon is driven by several fundamental mechanisms. Dysregulation of the cell cycle allows cancer cells to bypass normal checkpoints, resulting in uncontrolled proliferation and genomic instability, which significantly contributes to tumor progression [1,2]. Moreover, cancer cells can spread from the primary tumor to distant sites in a process known as metastasis, a major challenge in treatment and a leading cause of cancer-related deaths [3]. Additionally, tumors induce the formation of new blood vessels through angiogenesis, ensuring a sufficient supply of nutrients for continued growth while facilitating metastasis [4]

Over time, cancer treatment strategies have evolved from conventional approaches, such as chemotherapy and radiotherapy, to more targeted therapies [5]. Among the emerging molecular targets in cancer therapy is Heat Shock Protein 90 (HSP90), a molecular chaperone essential for the stability, folding, and activation of numerous oncogenic proteins [6]. HSP90 regulates key cellular signaling pathways involved in tumor growth, angiogenesis, and drug resistance by stabilizing receptor tyrosine kinases, transcription factors, and intracellular signaling proteins [7]. Given its crucial role in cancer progression, HSP90 has been widely explored as a promising therapeutic target. Its inhibition disrupts the stability of oncogenic proteins, leading to tumor cell death and reduced resistance to treatment [8].

Rosmarinus officinalis, commonly known as rosemary, has garnered significant attention for its potential anticancer properties [9]. Studies have demonstrated that various extracts and constituents of rosemary exert cytotoxic effects on different cancer cell lines, highlighting its potential as a natural therapeutic agent [10]. The anticancer mechanisms of rosemary include reducing cell viability in cancer cell lines such as KB and A549, with IC50 values indicative of effective therapeutic concentrations [11]. Additionally, rosemary extracts modulate the expression of cancer-associated genes, particularly by downregulating matrix metalloproteinase-9 (MMP-9) and tissue inhibitor of metalloproteinase-1 (TIMP-1), both implicated in cancer progression. Among its bioactive constituents, rosmarinic acid—a key polyphenol—exhibits potent anticarcinogenic properties, especially in gastrointestinal malignancies [12]. Other compounds, including camphor and α-pinene, contribute to rosemary’s anticancer and antioxidant activities [13]. Moreover, rosemary essential oil has demonstrated significant anticancer effects by reducing immunoreactivity associated with cancer proliferation in treated cells [14]. While these findings highlight the potential of rosemary as a complementary therapy in oncology, further investigation is necessary, particularly regarding its role in overcoming limitations associated with conventional chemotherapies.

In this study, we focus on evaluating rosemary as a potential inhibitor of HSP90. Our research employs an in silico approach, utilizing advanced computational techniques to predict the interactions between rosemary-derived compounds and HSP90. Through molecular interaction simulations and structural analysis, we aim to identify compounds with strong binding affinities to HSP90, thereby assessing their potential to inhibit this key oncogenic protein.

The use of in silico methods offers a cost-effective and time-efficient strategy for identifying potential therapeutic compounds before advancing to experimental validation. This computational approach accelerates the drug discovery process and facilitates the identification of novel anticancer agents. Additionally, network pharmacology provides a holistic perspective by analyzing the interactions of biological targets within complex protein–protein interaction (PPI) networks, enabling the identification of multitarget drugs with enhanced therapeutic efficacy and reduced resistance compared to single-target approaches [15].

Furthermore, molecular docking simulations allow for the prediction of binding affinities between candidate compounds and target proteins, providing crucial insights into complex stability and potential therapeutic efficacy [16]. The objective of this study is to explore the anticancer potential of rosemary-derived compounds by targeting HSP90 and other key proteins involved in cancer progression. By integrating network pharmacology with molecular docking simulations, we aim to identify the most promising rosemary compounds capable of modulating these targets. Specifically, by targeting HSP90, we seek to offer new insights into its inhibition as a potential strategy to destabilize cancer cells and enhance treatment outcomes, addressing key challenges posed by conventional cancer therapies.

## 2. Materials and Methods

### 2.1. Identification of Rosmarinus officinalis Compounds and Prediction of Their Biological Targets

The bioactive compounds of *R. officinalis* were retrieved from the scientific literature [17,18]. A total of 12 compounds were identified, with their chemical structures and specific properties summarized in Table 1. To predict their potential biological targets, we utilized the SwissTargetPrediction platform, which identified 506 potential target genes (Table S1). Only targets with a predicted probability score of ≥0.1 were considered for further analysis, in order to ensure a reliable confidence level and minimize false-positive predictions.

These data provide a strong foundation for investigating the mechanisms of action of *R. officinalis* compounds and their roles in various biological and pathological processes.

### 2.2. Identification of Genes Associated with Anti-Tumor Agents

To identify genes involved in anticancer mechanisms, we utilized the GeneCards database. This analysis identified a total of 1091 genes potentially contributing to tumor suppression. These genes represent a valuable resource for further exploration of molecular pathways and the identification of novel therapeutic targets. Tumor-suppressor-related genes (n = 1091) were retrieved from the GeneCards database using the keyword ‘tumor suppressor’, and results were ranked based on the GeneCards relevance score. The complete list is available in Supplementary Table S1.

Following the identification of common target proteins, further analyses were conducted to deepen the investigation. The tools used in these analyses are summarized in the table below (Table 2). The primary aim of GO enrichment and PPI network analysis (DAVID, STRING, Cytoscape, and ShinyGO) was to prioritize key targets based on their functional significance, pathway enrichment, and network centrality, thereby identifying the most biologically relevant genes for subsequent docking and molecular dynamics studies.

### 2.3. Optimization of Protein Structures and Ligands for Molecular Docking Accuracy

The optimization of protein structures and ligands was performed to ensure accurate docking analysis. Protein–ligand docking was conducted using Schrödinger Release 2020-2: Maestro, Schrödinger, LLC, New York, NY, 2020. The structure of the HSP90 protein (PDB ID: 3OWD) [19] a human molecular chaperone, was obtained from the Protein Data Bank. This structure, resolved by X-ray diffraction at a resolution of 2.90 Å, is complexed with an N-aryl-benzimidazolone inhibitor.

Protein preparation was carried out using the Protein Preparation Wizard tool (Schrödinger Release 2020-4) [20], which included hydrogen atom addition, protonation state adjustments, and necessary structural refinements. Ligands derived from *R. officinalis* were prepared using LigPrep2020 (Schrödinger Release 2020-4) [20], generating multiple possible conformations. Initial docking was performed using Glide in Standard Precision (SP) mode. Additional details on protein and ligand preparation methods are available in our previous studies [21].

The docking results were evaluated based on binding energy and molecular interactions, particularly hydrogen bonding and hydrophobic interactions. Further exploration of molecular interactions was performed using Discovery Studio 2024 (https://discover.3ds.com/discovery-studio-visualizer-download, accessed on 25 September 2024).

To validate the docking methodology, the Maestro enrichment calculation panel was employed. First, active compounds and decoys were prepared using LigPrep2020 (Schrödinger Release 2020-4). These compounds were then docked into the HSP90 protein using Glide SP mode. The active compounds, selected based on previous studies reporting moderate IC50 values against HSP90 [22,23], served as reference ligands. Decoys were generated from one of the active compounds using the PubChem database, applying a 20% structural similarity threshold. Both active and decoy compounds were formatted in SDF files for docking analysis.

### 2.4. ADMET and Drug-likeness Properties

The administration of a medicinal product to humans requires specific pharmacokinetic properties to ensure its absorption, distribution, metabolism, and excretion (ADME). Therefore, evaluating the safety and toxicity profiles of drug candidates using in silico ADMET approaches is a crucial step in pharmaceutical development. In this study, the ADMETlab 3.0 server [24] (accessed on 1 January 2025) was used to assess the ADMET properties of lead ligands. Additionally, the ProTox 3.0 server [25] was employed to evaluate toxicological parameters, including cytotoxicity, mutagenicity, carcinogenicity, and hepatotoxicity, providing a comprehensive risk assessment for their therapeutic application.

### 2.5. Molecular Dynamics (MD) Simulations

Molecular dynamics (MD) simulations were performed using Desmond software (Schrödinger Release 2020-4) (Schrödinger LLC, New York, NY, USA) [26] The simulations were conducted under the NPT ensemble, maintaining a temperature of 300 K and a pressure of 1 bar. The Martyna–Tuckerman–Klein coupling method (coupling constant: 2.0 ps) was used for pressure control, while the Nosé–Hoover method was applied for temperature regulation [27].

The atomic interactions were modeled using the OPLS_2005 force field, and long-range electrostatic forces were calculated using the particle mesh Ewald (PME) method with a Coulomb interaction cutoff radius of 9.0 Å [28]. Water molecules were represented using the Simple Point Charge (SPC) model [29]. Non-bonded forces were computed with short-range forces updated at every step and long-range forces updated every three steps. Each simulation was performed for 100 ns, with a relaxation time of 1 ps [30].

Data trajectories were recorded for further analysis, covering four protein–ligand complexes: L1-HSP90, L2-HSP90, L3-HSP90, and MEY-HSP90. The stability of these complexes was evaluated by analyzing Root Mean Square Deviation (RMSD), Root Mean Square Fluctuation (RMSF), and protein–ligand contacts throughout the simulation. These analyses were conducted using the Simulation Interaction Diagram (SID) (Schrödinger Release 2020-4) (Schrödinger LLC, New York, NY, USA, tool integrated into Desmond).

## 3. Results

### 3.1. Common Targets of Rosemary (Rosmarinus officinalis) and Antitumor Agents

**Figure 1:**
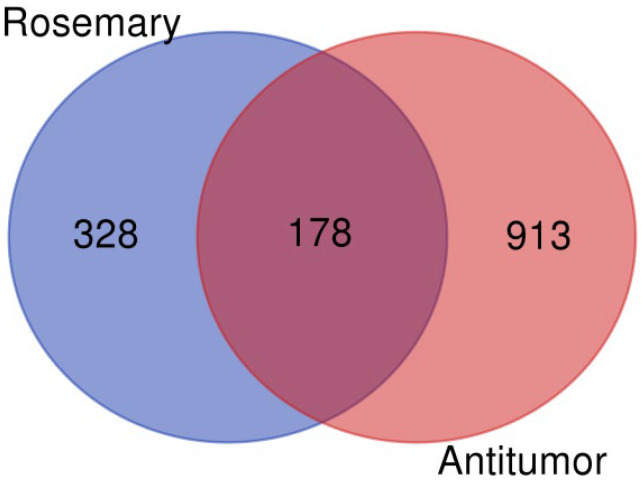
Venn Diagram Showing the Intersection of Predicted Target Proteins for *R. officinalis* (Rosemary) Compounds and Antitumor Target Protein.

As part of our investigation into the antitumor potential of *R. officinalis* (rosemary) compounds, we first compiled a list of target proteins associated with the 12 bioactive compounds extracted from this plant. These proteins were then cross-referenced with known proteins involved in antitumor mechanisms, which were retrieved from the GeneCards database (https://www.genecards.org/) using the keyword ‘tumor suppressor’ to identify relevant targets associated with cancer biology. This intersection yielded 178 proteins, as illustrated in Figure 1, generated using the VennDiagram website (https://bioinformatics.psb.ugent.be/webtools/Venn/, accessed on 18 September 2025). The full list of identified proteins is provided in Table S1.

To further analyze these proteins, we used the STRING database to study protein–protein interaction (PPIs) and construct an interaction network. This network was then exported to Cytoscape for visualization and in-depth analysis.

To refine our selection and identify the most biologically relevant proteins, we applied three key network analysis metrics: Mathew’s Correlation Coefficient (MCC)—Assesses the correlation strength between proteins, helping to identify those with robust and significant interactions in biological processes [31]. Betweenness Centrality—Measures a protein’s role in the network based on its ability to connect different nodes. Proteins with high Betweenness Centrality often serve as critical regulators of biological pathways [32]. Degree—Quantifies the number of direct interactions a protein has within the network. Proteins with high Degree values are frequently involved in essential cellular functions, making them promising therapeutic targets [33].

**Figure 2:**
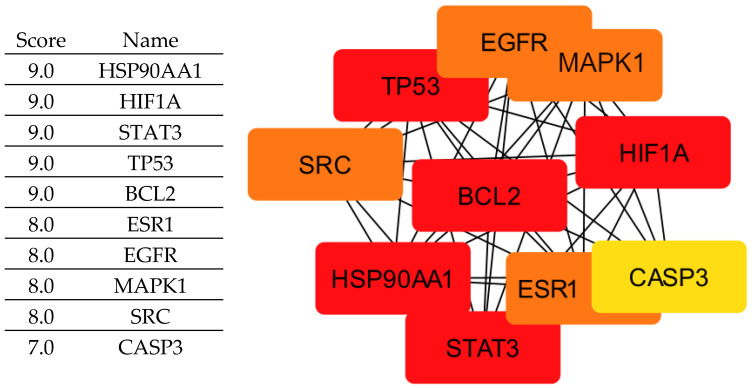
Core PPI Network of the Top 10 Cancer-Related Targets Identified by CytoHubba (Degree Method). The color gradient from red to yellow indicates the degree of node importance, with red representing the most significant hub genes and yellow representing those with lower significance.

By integrating these criteria, we identified ten key proteins (Figure 2) (EGFR, ESR1, HIF1A, HSP90AA1, MAPK1, BCL2, STAT3, TP53, CASP3, and SRC) that play central roles in tumor progression and are therefore considered crucial therapeutic targets for rosemary-derived compounds. It is important to clarify that not all of these proteins act exclusively as tumor suppressors; some, such as EGFR, STAT3, SRC, and HSP90AA1, are oncogenic drivers whose inhibition can suppress cancer growth. Others, like TP53 and CASP3, function as tumor suppressors but may be indirectly modulated or stabilized by the compounds rather than inhibited. Therefore, the compounds may exert their effects either by inhibiting oncogenic targets or by supporting and enhancing the activity of tumor-suppressive pathways. 

### 3.2. Enrichment Analysis of Key Proteins

**Figure 3:**
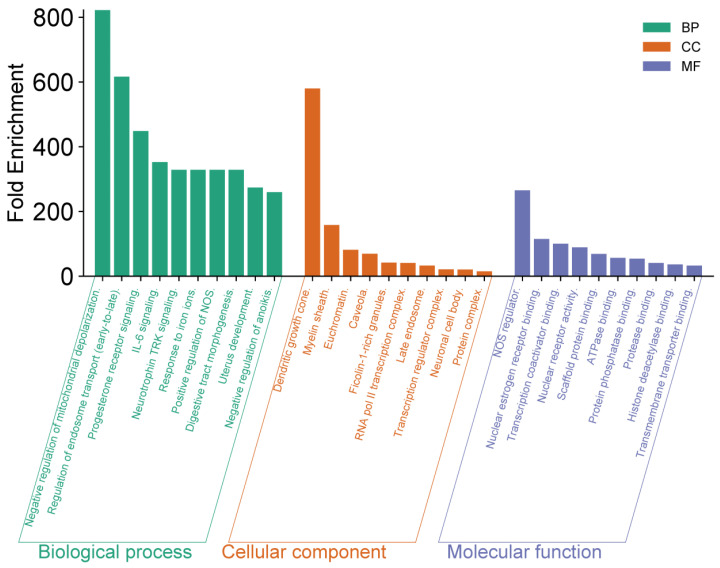
Enrichment analysis for HUBGENE was performed using DAVID. The top 10 GO terms with the most significant enrichment (*p* < 0.05) across the biological process, molecular function, and cellular component categories are presented.

To further investigate the 10 selected proteins (identified based on their high MCC, Betweenness Centrality, and Degree in the PPI network), we conducted functional annotation and enrichment analysis using the DAVID (Database for Annotation, Visualization, and Integrated Discovery) platform (https://davidbioinformatics.nih.gov/, accessed on 18 September 2025). This analysis allowed us to explore the biological functions, signaling pathways, and cellular processes associated with these proteins. The results, presented in Figure 3, highlight key biological functions and interactions that may contribute to the antitumor efficacy of *R. officinalis* compounds.

The functional enrichment analysis revealed several significant observations. At the biological process (BP) level, negative regulation of mitochondrial depolarization exhibited the highest enrichment, exceeding 800-fold. Other highly enriched processes included regulation of endosome transport and progesterone receptor signaling, both surpassing 400-fold enrichment. Regarding cellular components (CC), dendritic growth cone and myelin sheath showed remarkable enrichments, reaching values close to 350-fold and 300-fold, respectively. In terms of molecular function (MF), NOS regulation and nuclear receptor binding displayed high enrichment values, approaching 250-fold, indicating a crucial role in intracellular signal regulation. These findings highlight the potential role of rosemary-derived bioactive compounds in modulating key cellular processes associated with tumor development, and opening promising avenues for novel anticancer therapeutic strategies.

**Figure 4:**
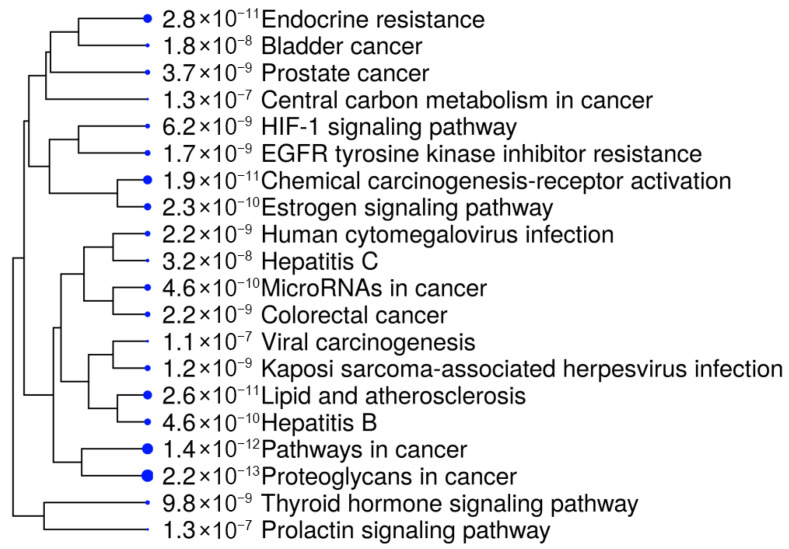
Gene Ontology (GO) enrichment analysis of the ten selected proteins. The figure highlights the most enriched biological processes, cellular components, and molecular functions, revealing key regulatory roles in cancer-related pathways.

To gain deeper insights, we further analyzed the ten selected proteins using the SHINY GO platform (https://bioinformatics.sdstate.edu/go/, accessed on 18 September 2025). The results, presented in Figure 4 and Table 3, demonstrated a significant involvement of these genes in several cancer-related biological and pathological pathways. Notably, the most enriched pathways included bladder cancer, resistance to EGFR tyrosine kinase inhibitors, and endocrine resistance, suggesting a strong association with tumor progression and drug resistance mechanisms [34,35].

Other relevant pathways included central metabolic pathways, prolactin signaling, and microRNAs involved in cancer, underscoring the regulatory role of the selected targets in tumorigenesis and cancer cell survival. Additionally, chemical carcinogenesis pathways and their involvement in Hepatitis B/C-related cancer development indicate the broader therapeutic potential of *R. officinalis* compounds in various oncogenic and pathological contexts. The high enrichment values observed in proteoglycans in cancer and estrogen signaling pathways further reinforce their potential as targeted cancer therapeutics.

Based on the enrichment analysis and pathway data presented in Table 3, we selected HSP90 (Heat Shock Protein 90) as the primary target for molecular docking studies. HSP90 exhibited the highest Degree score and was the most frequently implicated protein in cancer-related pathways, reinforcing its role as a key therapeutic target. These findings support the hypothesis that bioactive compounds from *R. officinalis* may exert anticancer effects by modulating HSP90 and other critical pathways, paving the way for future experimental validation and potential drug development.

### 3.3. Docking Study of Substances from R. Officinalis as Hsp90 Inhibitors

This study focused on the Hsp90 protein as the primary target for docking analysis with twelve bioactive compounds sourced from *R. officinalis* (rosemary). The aim was to investigate the molecular interactions between Hsp90 and rosemary-derived substances, particularly their potential as inhibitors. The three-dimensional structure of Hsp90 (PDB ID: 3OWD) was obtained from the Protein Data Bank (PDB) (https://www.rcsb.org/structure/3OWD, accessed on 10 October 2024). The active site was identified based on the reference compound, Mey (N-{[1-(5-chloro-2,4-dihydroxyphenyl)-2-oxo-2,3-dihydro-1H-benzimidazol-5-yl]methyl}naphthalene-1-sulfonamide), which had previously been docked into the crystalline structure. The principal amino acids forming the active site include a hydrophobic component, consisting of residues Ala55, Ile96, Met98, Leu107, Phe138, and Val150, and a hydrophilic component, comprising residues Asn51, Asp93, and Thr184. These findings are consistent with previous studies [23,36].

To ensure the reliability of the docking method, we first validated its precision. During this validation phase, we used 35 active compounds known to inhibit Hsp90 and 1000 inactive compounds sourced from the PubChem database. Several statistical metrics were calculated to assess the docking procedure, including ROC (Receiver Operating Characteristic curve), BEDROC (Boltzmann-enhanced discrimination of ROC), AUC (Area Under the Curve), and RIE (Robust Initial Enhancement), as detailed in Tables S2–S5.

**Figure 5:**
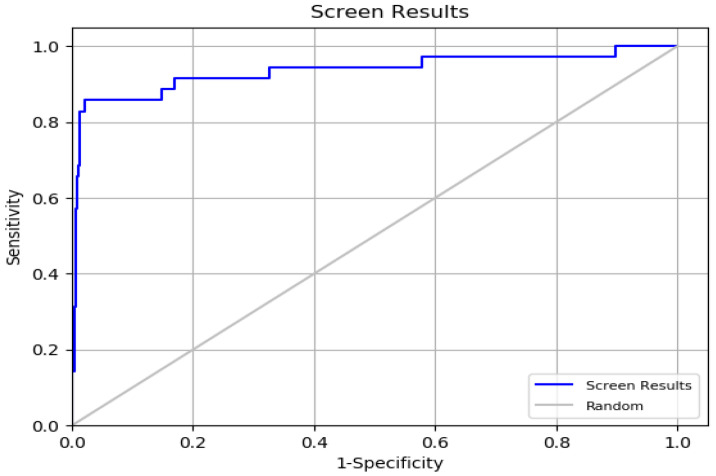
Enrichment Curve for Virtual Screening Results.

The ROC value, which ranges from 0 to 1, was found to be 0.93, indicating strong efficacy in ranking active compounds above inactive ones [37]. This was further supported by the ROC plot in Figure 5. The AUC value of 0.92 shows that 92% of real positive findings were correctly identified by the docking method, underscoring its effectiveness in distinguishing active compounds from decoys. The RIE value was determined to be 11.54, a favorable outcome for validation. The BEDROC values at various tuning parameters were (α = 8.0/0.845), (α = 20.0/0.794), and (α = 160.9/0.657). The BEDROC metric indicates the probability that an active molecule is ranked higher than inactive compounds [38]. These results confirm the reliability and effectiveness of the docking method for virtual screening.

Following validation via enrichment analysis, which yielded significant enrichment of potential therapeutic targets, we initiated molecular docking simulations to investigate the binding interactions between bioactive constituents of *R. officinalis* and the ATP-binding domain of heat shock protein 90 (HSP90; PDB ID: 3OWD). These computational studies were designed to elucidate the molecular mechanisms underlying the potential inhibitory effects of the compounds on HSP90 function. The binding affinities, quantified as docking scores (XP GScore) for each derivative, are detailed in Table 4.

**Figure 6:**
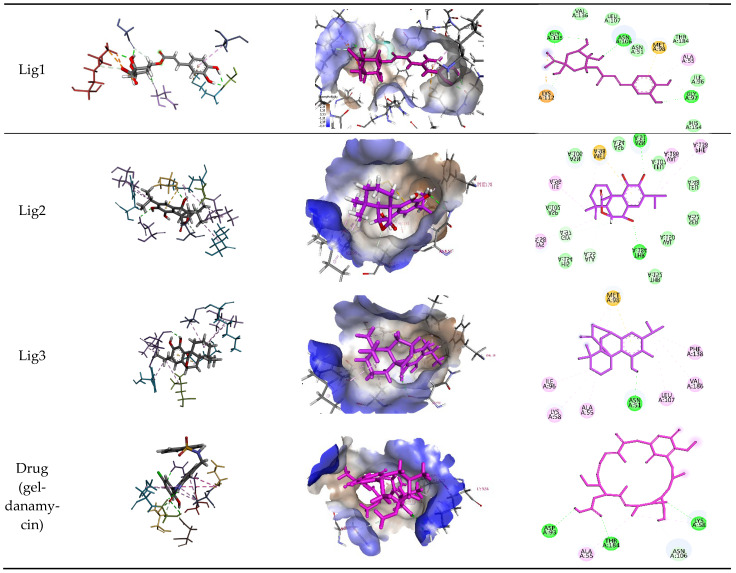
2D and 3D Interaction Representations of the Top Docking Compounds with HSP90 Protein and the Reference Drug Geldanamycin.

To benchmark the binding efficacy of the *R. officinalis* derivatives, molecular docking results were compared with two established reference inhibitors: (i) the co-crystallized ligand Mey (PDB: 3OWD), which serves as a structural and energetic benchmark for the HSP90 active site, and (ii) Geldanamycin (DB02424), a clinically validated HSP90 inhibitor. The docking scores (XP GScore) revealed distinct binding affinities: Mey exhibited a score of −7.25 kcal/mol, consistent with its moderate inhibitory potency, while Geldanamycin displayed a weaker interaction (−5.432 kcal/mol), likely reflecting its distinct binding mode or dynamic instability within the rigid docking framework. Strikingly, the tested derivatives demonstrated superior binding energetics, with Lig 1 achieving the highest affinity (−9.762 kcal/mol), followed by Lig 2 (−7.317 kcal/mol) and Lig 3 (−7.280 kcal/mol). These results suggest that Lig 1, in particular, may occupy the HSP90 binding pocket with enhanced complementarity compared to both reference compounds. Analysis of the ligand-protein interaction networks demonstrated that the bioactive derivatives form critical intermolecular contacts—including hydrogen bonds with conserved residues (e.g., Asp93, Asn51) and hydrophobic interactions with the ATP-binding pocket—mirroring the binding patterns observed for the reference inhibitors Mey and Geldanamycin (Figure 6 and Table 5).

Chlorogenic Acid (Lig 1) possesses a structure rich in hydroxyl groups (-OH), which contribute significantly to hydrogen bonding, both as donors and acceptors. This explains its formation of eight hydrogen bonds with residues such as Asn106, Gly97, Gly135, and Lys112. These interactions indicate a high affinity for the polar regions of the active site. However, the limited number of hydrophobic interactions (only two, involving Ala55 and Lys112) may reduce its stability in the hydrophobic pockets of HSP90, which play a crucial role in ligand docking. Consequently, despite strong polar interactions, Chlorogenic Acid may be less effective in engaging with the more hydrophobic regions of the protein.

Rosmanol (Lig 2) demonstrates a well-balanced interaction profile between hydrogen and hydrophobic interactions due to its diterpenic structure, which contains multiple rings and functional groups such as carbonyls and hydroxyls. It forms four hydrogen bonds with key residues like Asn51, Gly97, and Thr184, while also establishing seven hydrophobic interactions with residues such as Ile96, Lys58, Met98, and Phe138. These hydrophobic interactions enhance Rosmanol’s affinity for the non-polar regions of the active site, particularly around Met98, which appears to play a pivotal role in stabilizing this ligand.

Carnosol (Lig 3), structurally similar to Rosmanol, displays a different binding behavior. It forms only one hydrogen bond (with Asn51), suggesting a lower affinity for the polar regions of the protein. However, it compensates for this with seven strong hydrophobic interactions involving Ala55, Ile96, Leu107, Lys58, Met98, and Phe138. The repeated involvement of Met98 as a key interacting residue for both diterpene ligands highlights its importance in ligand recognition and anchoring within the hydrophobic pockets of HSP90.

In comparison, the reference compound Geldanamycin exhibits a balanced profile, forming six hydrogen bonds with residues such as Asn106, Asp93, and Thr184, along with two hydrophobic interactions (Ala55 and Lys58). Although its hydrophobic interactions are moderate compared to the natural ligands, its well-distributed hydrogen bonds contribute to its established efficacy as an HSP90 inhibitor. However, its weaker performance in hydrophobic pockets suggests that the natural rosemary-derived ligands may offer superior stability and affinity.

These results suggest that the hydrophobic, ring-rich structures of natural ligands, such as Rosmanol and Carnosol, confer a significant advantage in terms of stability and affinity for HSP90. Multiple interactions with key hydrophobic residues, including Met98, Ile96, and Phe138, reinforce their ability to anchor firmly within the active site. Conversely, while Chlorogenic Acid demonstrates strong interactions with polar residues, it appears less suited for engaging with the critical hydrophobic pockets of HSP90.

### 3.4. Prediction of ADMET Profiles and Drug-likeness of the Lead Ligands

The evaluation of ADMET (Absorption, Distribution, Metabolism, Excretion, and Toxicity) properties is crucial for predicting and mitigating potential risks associated with the administration of new therapeutic molecules. This analysis provides key insights to minimize failures during clinical trials by assessing the pharmacokinetic and safety profiles of drug candidates.

In this study, we examined the ADMET profiles of the identified lead ligands (Lig 1, Lig 2, and Lig 3), focusing on their pharmacokinetic behavior, toxicity, drug-likeness, and physicochemical properties. These assessments help determine the compounds’ potential efficacy and safety as drug candidates, ensuring they meet the necessary criteria for further pharmaceutical development.

#### 3.4.1. Physicochemical Properties

Table 6 presents the physicochemical and medicinal properties of the lead ligands, which exhibit molecular weights and volumes within an optimal range (330–355 Da), lower than that of Geldanamycin. This suggests their enhanced potential for favorable interactions with HSP90 and promising therapeutic characteristics.

The Total Polar Surface Area (TPSA) is a crucial parameter influencing a molecule’s ability to penetrate biological membranes and be effectively absorbed by the body [39]. Compounds with a TPSA exceeding 140 Å^2^ generally exhibit poor membrane permeability, whereas those with TPSA values ≤ 60 Å^2^ are typically well absorbed. The analysis indicates that Lig 2 and Lig 3 have TPSA values within the optimal range, i.e., below 140 Å^2^, which supports their favorable absorption and oral bioavailability. In contrast, Lig 1 possesses a significantly higher TPSA, surpassing the ideal threshold, which could compromise its gastrointestinal absorption and limit its oral therapeutic efficacy. This finding underscores the importance of optimizing physicochemical properties to improve pharmacokinetic profiles.

Regarding solubility (Log S), all ligands meet the required criteria, except for Lig 3, which slightly exceeds the optimal range (−4 to 0.5), potentially affecting its membrane permeability. The octanol–water partition coefficient (LogP) values of Lig 1 and Lig 2 fall within the ideal range (0–3), indicating a balanced hydrophilicity-lipophilicity profile and high bioavailability potential. Conversely, although the LogP of Lig 3 remains within an acceptable range, optimization may further enhance its pharmacokinetic properties and therapeutic efficacy.

In drug discovery, adherence to established guidelines is essential for identifying compounds with high therapeutic potential while ensuring safety and efficacy. Several key principles contribute to this selection process, including Lipinski’s Rule of Five [40], the Pfizer Rule, the GSK Rule [41], and the Golden Triangle Concept [42,43]. Lipinski’s Rule of Five establishes key structural attributes that favor oral bioavailability, such as a molecular weight below 500 Da, LogP under 5, and defined limits on hydrogen bond donors and acceptors. The Pfizer Rule [44], also known as the “3/75 Rule,” highlights potential toxicological risks associated with high lipophilicity (LogP greater than 3) and reduced TPSA (below 75 Å^2^). GSK’s Rule suggests that compounds with a molecular weight above 400 Da and LogP exceeding 4 may present safety and pharmacokinetic concerns. The Golden Triangle Concept integrates molecular weight, lipophilicity, and TPSA into an optimal range to balance efficacy, bioavailability, and safety [21]. These principles serve as critical selection criteria to streamline drug candidate identification and minimize development risks.

The synthetic accessibility assessment (SA) of the ligands yields values below 6, suggesting that their synthesis is feasible and straightforward. According to Table 6, Lig 1 and Lig 2 comply with the Lipinski, Pfizer, and GSK rules, as well as the Golden Triangle criteria. These findings indicate a high bioavailability potential, favorable pharmacokinetics, and promising therapeutic efficacy, surpassing that of Geldanamycin, which adheres only to the Lipinski and Pfizer rules, thereby limiting its pharmacological potential.

#### 3.4.2. Absorption

The results presented in Table 7 show notable differences between the compounds in terms of their absorption and membrane permeability. Regarding human intestinal absorption (HIA), Lig1 (Chlorogenic Acid), Lig2 (Rosmanol), and Lig3 (Carnosol) all exhibit values above 30%, indicating their potential for efficient absorption from the gastrointestinal tract. This suggests that these compounds may be suitable for oral administration. In contrast, Geldanamycin has an HIA value below 30%, which indicates limited absorption and may hinder its efficacy as an oral drug.

When considering Caco-2 permeability, a critical parameter for predicting a compound’s ability to cross intestinal membranes, lower values (negative and close to zero) are associated with reduced permeability. Lig1 shows the lowest permeability (−6.426 cm/s), consistent with its highly polar structure that is rich in hydroxyl groups. On the other hand, Lig3 (−4.767 cm/s) and Lig2 (−5.256 cm/s) display significantly better permeability, which can be attributed to their lipophilic nature due to their hydrophobic diterpene structures. These compounds are thus more likely to cross biological membranes with ease.

Geldanamycin, with a Caco-2 permeability value of −5.246 cm/s, falls between Lig2 and Lig1 but still performs worse than Lig3. This difference could help explain why Geldanamycin has a lower overall bioavailability compared to the natural compounds derived from rosemary.

#### 3.4.3. Distribution

Table 7 shows that all ligands exhibit low penetration through the blood–brain barrier (BBB) (<0.2), indicating limited potential for interaction with the central nervous system (CNS). Lig1 (Chlorogenic Acid) has a binding potential to plasma proteins (PPB) of less than 90%, which is similar to that of Geldanamycin, whereas the other ligands have higher PPB values (greater than 90%), suggesting a potentially limited systemic bioavailability for these compounds.

#### 3.4.4. Metabolism and Excretion

Regarding metabolism and excretion, none of the compounds evaluated exhibit significant inhibition of major cytochrome P450 (CYP) enzymes [45], which suggests a low risk of drug–drug interactions related to CYP inhibition. Lig2 (Rosmanol) and Lig3 (Carnosol) show moderate renal clearance values (5–15 mL/min/kg), with values of 12.456 and 12.709, respectively. In contrast, Lig1 has the lowest renal clearance value, indicating potentially reduced renal elimination. All ligands display relatively short half-lives (T½ < 3 h), with Lig3 having the shortest half-life, indicating faster elimination compared to Lig1 and Lig2.

#### 3.4.5. Toxicity

In terms of toxicity, the results from the ADMETlab 3.0 server reveal that the three ligands have low hERG scores (<0.1), indicating no significant potential for inhibiting the hERG ion channel. This is consistent with the behavior of Geldanamycin and suggests that these ligands do not present a risk of cardiotoxicity, which is crucial for ensuring the safety of drug candidates [46]. The mutagenic potential of Lig1 and Lig2 is considered moderate (0.3–0.7), while Lig3 is categorized as toxic. Similarly to Geldanamycin, the selected ligands exhibit a high potential for skin sensitization.

Further analysis using the Protox 3.0 platform (Table 8) suggests that the compounds evaluated have a low potential for toxicity, including hepatotoxicity, carcinogenicity, mutagenicity, and cytotoxicity. Geldanamycin does not show hepatotoxic, carcinogenic, or mutagenic potential but presents a moderate risk for cytotoxicity with an estimated probability of 0.67.

The ADMET properties and toxicity profiles of Lig3 highlight some limitations that may pose barriers to its use. These findings suggest the need for structural modifications to reduce potential risks and improve the safety and efficacy of Lig3 as a therapeutic candidate.

### 3.5. Molecular Dynamics (MD) Studies

The molecular dynamics (MD) simulations were conducted not only to validate the docking results but also to assess the stability, flexibility, and dynamic behavior of the ligand–HSP90AA1 complexes over time. This step is critical for understanding whether the predicted binding poses remain stable under physiological conditions and whether any significant conformational changes occur during the interaction.

In this analysis, the complexes (Lig1-Hsp90), (Lig2-Hsp90), and (Lig3-Hsp90), proposed as potential inhibitors of the HSP90 protein, were compared to the reference complex (MEY-Hsp90). The comparison was based on parameters such as RMSD (Root Mean Square Deviation), RMSF (Root Mean Square Fluctuation), and interactions with key residues, which are critical for assessing the stability and binding efficacy of these ligands to HSP90.

**Figure 7:**
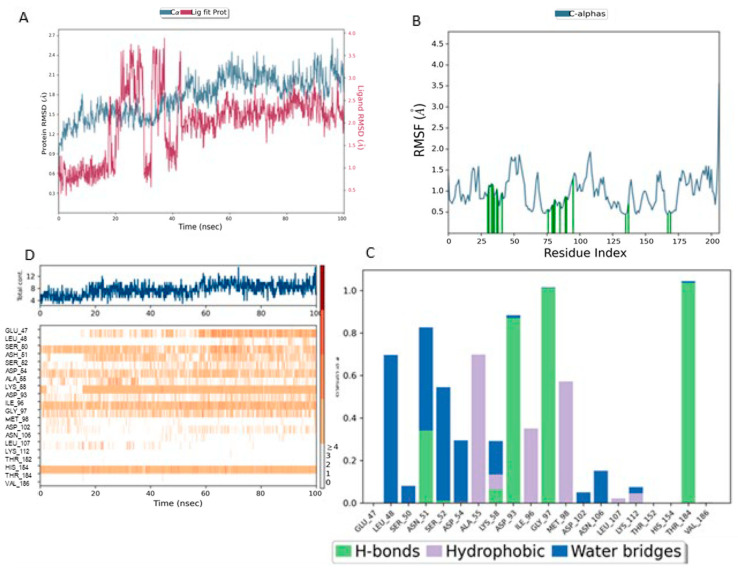
MD Simulation Data of Mey Interacting with the Hsp90 Protein (PDB ID: 3OWD). (**A**) RMSD of the Mey-Hsp90 Complex. Cα (blue): represents the protein backbone atoms (alpha-carbons), shown in blue to illustrate the overall fold of the protein. Lig fit Prot (red/pink): the protein structure (Prot) shown in red/pink is aligned and fitted based on the ligand (Lig) conformation (**B**) RMSF of the Mey-Hsp90 Complex. (**C**) Types of Interactions in the Mey-Hsp90 Complex. (**D**) Number of Contacts in the Mey-Hsp90 Complex.

**Figure 8:**
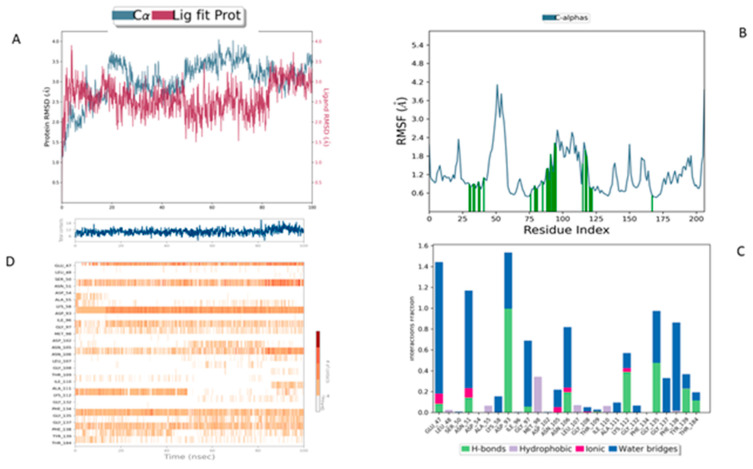
MD simulation data of Lig1 interacting with the Hsp90 protein (PDB ID: 3OWD). (**A**) RMSD of the Lig1-Hsp90 complex. Cα (blue): represents the protein backbone atoms (alpha-carbons), shown in blue to illustrate the overall fold of the protein. Lig fit Prot (red/pink): the protein structure (Prot) shown in red/pink is aligned and fitted based on the ligand (Lig) conformation (**B**) RMSF of the Lig1-Hsp90 complex. (**C**) Types of interactions in the Lig1-Hsp90 complex. (**D**) Number of contacts in the Lig1-Hsp90 complex.

RMSD (Root Mean Square Deviation) measures the average structural deviation and serves as a key indicator of complex stability. The MEY-Hsp90 complex (Figure 7A), considered as a reference, shows moderate stability throughout the simulation. The protein RMSD (blue curve) fluctuates between 1.6 and 3.2 Å, with a gradual increase beyond 50 ns, suggesting global structural adjustments in the protein due to its interaction with the MEY ligand. In contrast, the ligand RMSD (red curve) remains highly stable, with an average value of around 1.4 Å, indicating that its position and orientation remain relatively constant despite some local fluctuations. These results highlight the strong affinity and stable interaction of the MEY ligand with Hsp90, justifying its use as a reference for further studies. Figure 8 presents a detailed analysis of the molecular dynamics of the complex between Lig1 (Chlorogenic Acid) and the Hsp90 protein, highlighting its stability and key interactions. Plot (A) shows the evolution of the RMSD (Root Mean Square Deviation) of the protein (blue) and the ligand (red) over 100 ns. The RMSD of the protein fluctuates between 2 and 3.5 Å, indicating some flexibility, especially in specific regions. Meanwhile, the RMSD of the ligand remains more stable (~1–2 Å), suggesting that it stays well anchored in the active site without significantly disengaging.

**Figure 9:**
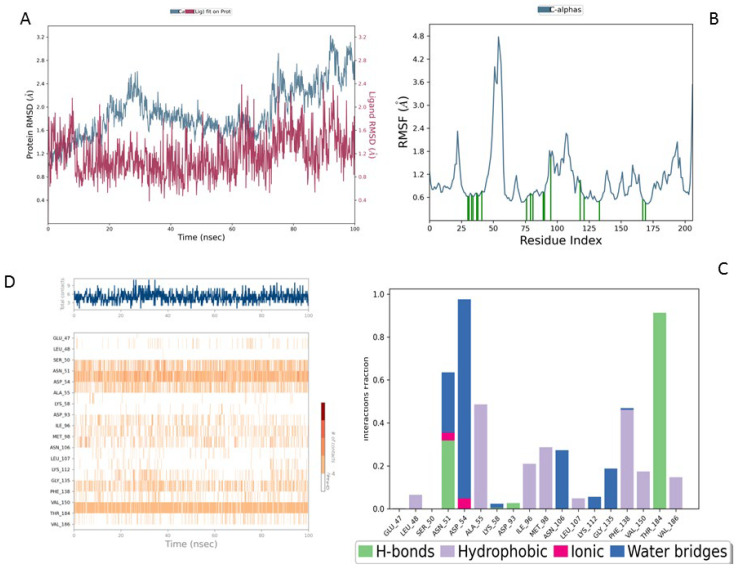
MD simulation data of Lig2 interacting with the HSP90 protein (PDB ID: 3OWD). (**A**) RMSD of the Lig2-HSP90 complex. (**B**) RMSF of the Lig2-HSP90 complex. (**C**) Types of interactions in the Lig2-HSP90 complex. (**D**) Number of contacts in the Lig2-HSP90 complex.

In comparison, the Lig2-HSP90 complex (Figure 9A), representing Lig2 (Rosmanol) derived from Rosemary, shows improved stability, particularly for the ligand. The protein RMSD reaches similar values to the reference complex, with an oscillation between 1.2 and 2.8 Å over the 100 ns. However, the stability of the ligand is more pronounced, with fluctuations limited to a range below 1.6 Å, indicating a better fit in the binding cavity. This enhanced stability may reflect increased hydrophobic or hydrogen interactions, highlighting a potentially increased affinity compared to the MEY ligand.

**Figure 10:**
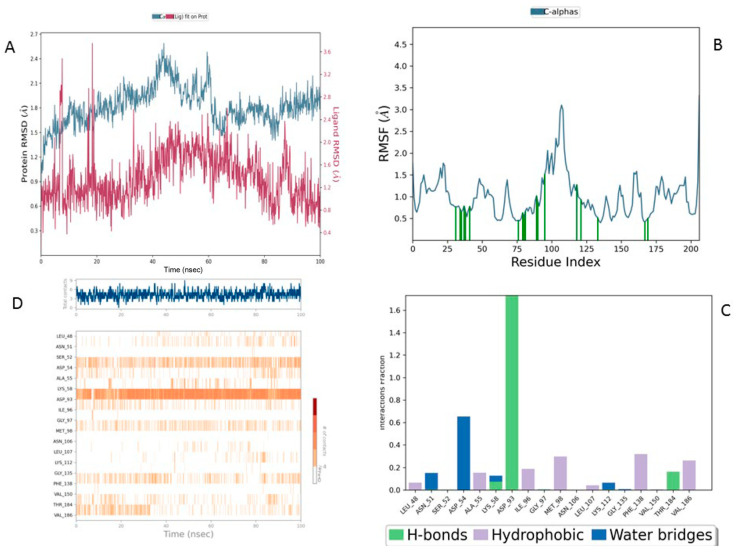
MD simulation data of Lig3 interacting with the HSP90 protein (PDB ID: 3OWD). (**A**) RMSD of the Lig3-HSP90 complex. Cα (blue): represents the protein backbone atoms (alpha-carbons), shown in blue to illustrate the overall fold of the protein. Lig fit Prot (red/pink): the protein structure (Prot) shown in red/pink is aligned and fitted based on the ligand (Lig) conformation (**B**) RMSF of the Lig3-HSP90 complex. (**C**) Types of interactions in the Lig3-HSP90 complex. (**D**) Number of contacts in the Lig3-HSP90 complex.

The Lig3-HSP90 complex (Figure 10A), on the other hand, shows even more promising performance. The protein RMSD remains on average lower than that of the MEY-HSP90 and Lig2-HSP90 complexes, stabilizing around 2.1 Å after 50 ns. This overall stability suggests less perturbation of the protein structure, which may be beneficial for efficient interaction. The ligand, in turn, exhibits very low RMSD fluctuations, staying mostly below 1.6 Å. This enhanced stability could be attributed to additional specific interactions or better conformational compatibility with the active site of HSP90.

Overall, RMSD analysis confirms that all three *R. officinalis* compounds form structurally stable complexes with HSP90, with Lig3 showing the most consistent behavior. This high degree of stability is indicative of effective binding and supports its potential as a lead compound for further investigation. Following the RMSD analysis, we next examined the RMSF profiles to identify flexible and stable regions and to pinpoint key residues involved in ligand interactions.

The reference MEY–HSP90 complex (Figure 7B) shows moderate overall stability, with fluctuations not exceeding 2.5 Å, indicating a relatively rigid backbone. Interaction analysis (Figure 7C) reveals moderate involvement of residues such as Leu41 and Glu47, with interaction frequencies around 0.6 and 0.5, respectively. The contact heat map (Figure 7D) displays a uniform interaction distribution with no concentrated hot spots, suggesting that while functional, the reference ligand may not fully optimize key binding interactions.

For the Lig1–HSP90 complex (Figure 8B), the RMSF profile shows distinct flexibility peaks around residues 50–75 and 100–125, which likely correspond to loop regions involved in ligand accommodation. Residues highlighted in green represent direct ligand interactions, confirming their contribution to stabilizing the complex. Interaction frequency analysis (Figure 8C) shows hydrophobic contacts (blue) as the dominant stabilizing force, complemented by stable hydrogen bonds (green) with Asn51, Ala55, Asp93, and Tyr139. Ionic and π–π stacking interactions (red and purple) further enhance binding affinity. The contact map (Figure 8D) demonstrates a consistent 10–15 contacts throughout the simulation, indicating robust and sustained ligand binding.

The Lig2–HSP90 complex (Figure 9B) displays slightly increased dynamic flexibility, with RMSF peaks reaching 4.8 Å at Asp54 and Val186, suggesting adaptive motion that may improve ligand accommodation. These residues also exhibit high interaction frequencies (~0.9 and 0.8) (Figure 9C,D), and the heat map shows prominent hot spots, underscoring stronger ligand–protein affinity compared to the reference complex.

The Lig3–HSP90 complex (Figure 10B) demonstrates superior overall performance. RMSFs remain low across most regions, except for Asp95 and Gly97, which show peaks of ~4.5 Å (Figure 10C,D), indicating localized flexibility that may facilitate binding. These residues exhibit very high interaction frequencies (up to 1.6), highlighting their critical roles in ligand stabilization. The heat map confirms these observations, displaying dense clusters and well-defined hot spots, which illustrate enhanced stabilization and strong ligand–protein interactions.

Collectively, these findings show that Lig2 and Lig3 form stronger and more persistent interactions with HSP90 than the reference ligand. Together with the RMSD results, this confirms that rosemary-derived compounds form stable, specific, and dynamically favorable interactions with HSP90AA1 under near-physiological conditions, providing strong computational evidence of their potential as effective inhibitors.

Beyond the computational data presented above, rosemary-derived phytochemicals—rosmanol, carnosol, and chlorogenic acid—have been extensively reported for their anticancer activities in diverse cancer models. These compounds modulate key signaling pathways, including PI3K/AKT, NF-κB, Wnt/β-catenin, and p53/p21, leading to apoptosis induction, cell cycle arrest, inhibition of metastasis, and suppression of tumor progression. Evidence from numerous in vitro and in vivo studies provides strong biological context to our computational predictions and further supports the rationale for targeting HSP90AA1 with these natural compounds.

While rosmanol, carnosol, and chlorogenic acid are known phytochemicals with previously reported anticancer properties, the novelty of this study lies in its integrative computational approach and its specific focus on HSP90AA1 as a molecular target—a critical chaperone in oncogenic signaling that has not been systematically explored in relation to these compounds. Unlike previous studies, which primarily investigated their general anticancer effects, our work combines network pharmacology, molecular docking, ADMET profiling, and molecular dynamics simulations to provide a detailed mechanistic and structural rationale for their potential as multi-target HSP90 inhibitors. This holistic approach not only offers new insights into how dietary phytochemicals can modulate HSP90-driven cancer pathways but also lays a strong foundation for future experimental validation and translational research.

## 4. Conclusions

This study deciphers the anticancer mechanisms of *R. officinalis* (rosemary) using an integrative computational approach combining network pharmacology, molecular docking, and molecular dynamics simulations. Network analysis identified three bioactive compounds—rosmanol, chlorogenic acid, and carnosol—as key mediators targeting HSP90, a molecular chaperone critical for oncogenic signaling and tumor progression. Protein–protein interaction (PPI) networks and functional enrichment were used to prioritize biologically relevant targets and pathways, revealing their polypharmacological effects on signaling cascades such as PI3K-AKT, apoptosis, and NF-κB-mediated immune regulation.

Pharmacokinetic assessments confirmed drug-likeness compliance (Lipinski’s Rule), moderate blood–brain barrier (BBB) penetration (reducing neurotoxic risks), and favorable absorption/distribution profiles. Toxicity predictions indicated no mutagenicity (Ames test) and limited CYP450 interactions, suggesting a safer profile compared to conventional inhibitors like geldanamycin.

Molecular dynamics simulations (100 ns) demonstrated stable binding of all three compounds to HSP90, with rosmanol showing the highest stability (RMSD < 3.0 Å). These simulations were performed to validate docking results and to assess the stability, dynamic behavior, and conformational impact of ligand binding over time. Structural analyses revealed persistent hydrogen bonds with catalytic residues (Asp93, Asn51) and hydrophobic stabilization in the ATP-binding pocket, consistent with competitive inhibition. Critically, the ligands preserved HSP90’s native conformation, indicating selective binding without allosteric destabilization—a key advantage over classical ATP-competitive inhibitors.

These findings highlight rosemary-derived compounds as multi-target HSP90 inhibitors with dual modulation of immune and apoptotic pathways, aligning with natural product-based drug discovery paradigms. However, as this study is based on in silico approaches, future work should include in vitro and in vivo validation—such as evaluating the effects of these compounds on cancer cell proliferation and HSP90 activity—along with bioavailability optimization and structural refinement to enhance selectivity. This study provides a framework for repurposing phytochemicals as adjuvants or combinatorial agents in precision oncology.

## Figures and Tables

**Table 1:** Summary of Compounds Found in Rosemary (*Rosmarinus officinalis*).

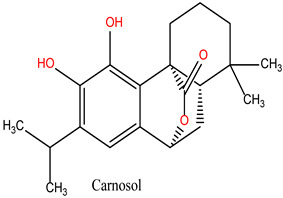	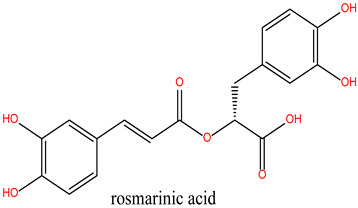	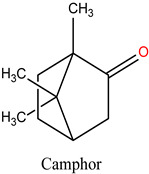
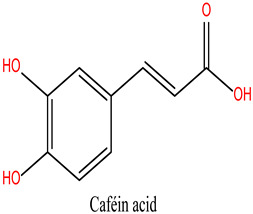	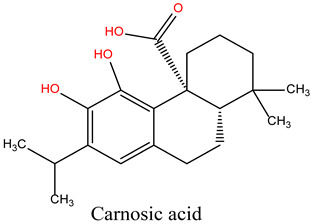	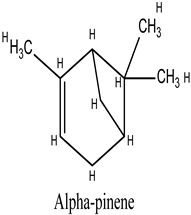
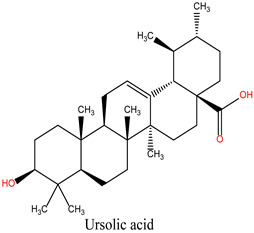	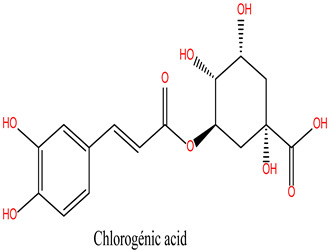	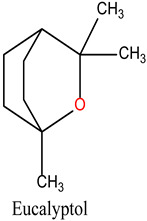
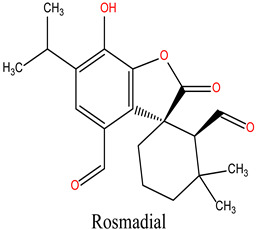	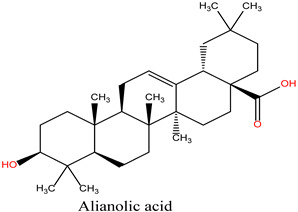	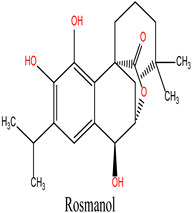

**Table 2:** Tools and Databases for Protein Analysis and Enrichment Studies.

Tool	Objective	Link
STRING	Analysis of protein structural interactions.	https://string-db.org/ (accessed on 14 October 2025)
Cytoscape	Visualization and analysis of biological networks.	https://cytoscape.org/ (accessed on 14 October 2025)
DAVID	Functional annotation and gene enrichment analysis.	https://davidbioinformatics.nih.gov/ (accessed on 14 October 2025)
Bioinformatics	Bioinformatics Data Analysis	https://www.bioinformatics.com.cn/en/ (accessed on 14 October 2025)
ShinyGO	Enrichment analysis and exploration of biological pathways.	http://bioinformatics.sdstate.edu/go/ (accessed on 14 October 2025)

**Table 3:** Pathway Analysis: Gene Count, Enrichment, and Associated Pathways.

nGenes	Pathway Genes	Fold Enrichment	Pathway
4	41	223.22	Path:hsa05219 Bladder cancer
5	79	144.81	Path:hsa01521 EGFR tyrosine kinase inhibitor resistance
6	95	144.51	Path:hsa01522 Endocrine resistance
5	86	133.02	Path:hsa05210 Colorectal cancer
4	70	130.74	Path:hsa04917 Prolactin signaling pathway
4	70	130.74	Path:hsa05230 Central carbon metabolism in cancer
5	97	117.94	Path:hsa05215 Prostate cancer
5	109	104.95	Path:hsa04066 HIF-1 signaling pathway
6	138	99.48	Path:hsa04915 Estrogen signaling pathway
5	121	94.54	Path:hsa04919 Thyroid hormone signaling pathway
8	202	90.61	Path:hsa05205 Proteoglycans in cancer
6	161	85.27	Path:hsa05206 MicroRNAs in cancer
6	162	84.74	Path:hsa05161 Hepatitis B
7	197	81.30	Path:hsa05207 Chemical carcinogenesis-receptor activation
7	214	74.84	Path:hsa05417 Lipid and atherosclerosis
5	157	72.86	Path:hsa05160 Hepatitis C
6	194	70.76	Path:hsa05167 Kaposi sarcoma-associated herpesvirus infection
6	224	61.28	Path:hsa05163 Human cytomegalovirus infection
5	202	56.63	Path:hsa05203 Viral carcinogenesis
9	530	38.85	Path:hsa05200 Pathways in cancer

**Table 4:** Docking Scores of *R. officinalis* Derivatives with the HSP90 Protein.

Ligands	Name	CID	Docking Score Kcal/Mol
Lig 1	Chlorogenic Acid	1794427	−9.762
Lig 2	Rosmanol	13966122	−7.317
Lig 3	carnosol	442009	−7.280
Lig 4	rosmarinic acid	5281792	−7.099
Lig 5	rosmadial	15801061	−6.078
Lig 6	Oleanolic Acid	10494	−6.078
Lig 7	Carnosic acid	65126	−6.035
Lig 8	caffeic acid	689043	−5.630
Lig 9	Ursolic acid	64945	−4.969
Lig 10	camphor	2537	−2.889
Lig 11	Alpha-Pinene	6654	−2.878
Lig 12	Eucalyptol	2758	−2.756
Reference Ligand	MEY	50925477	−7.25
Drug	Geldanamycin	5288382	−5.432

**Table 5:** Interaction Results of Top Docking Compounds with HSP90 Protein and the Drug (geldanamycin).

Com	NAME	H-bond	N°	Hydrophobic	N°	Other
Lig 1	Chlorogenic Acid	Asn106 Gly97 Gly135 Lys112	8	Ala55 Lys112	2	Lys112 Met98
Lig 2	Rosmanol	Ala55 Asn51 Gly97 Thr184	4	Ile96 Lys58 Met98 Phe138 Val186	7	Met98
Lig 3	Carnosol	Asn51	1	Ala55 Ile96 Leu107 Lys58 Met98 Phe138 Val186	7	Met98
Drug	(geldanamycin)	Asn106 Asp93 Lys58 Thr184	6	Ala55 Lys58	2	////

**Table 6:** Physicochemical and medicinal properties of the lead ligands and geldanamycin.

Ligands	Lig 1	Lig 2	Lig 3	Geldanamycin
Physicochemical Properties				
MW	354.1	346.18	330.18	560.27
Volume	331.473	353.656	344.865	570.405
nHA	9.0	5.0	4.0	11.0
nHD	6.0	3.0	2.0	4.0
TPSA	164.75	86.99	66.76	163.48
logS	−2.958	−3.941	−4.077	−2.874
logP	1.036	2.63	3.11	1.444
Medicinal Properties				
SAscore	3.0	5.0	5.0	5.0
Lipinski Rule	Accepted	Accepted	Accepted	Accepted
Pfizer Rule	Accepted	Accepted	Rejected	Accepted
GSK Rule	Accepted	Accepted	Accepted	Rejected
Golden Triangle	Accepted	Accepted	Accepted	Rejected

Note: Molecular weight (MW) below 500 Da indicates good membrane permeability and drug-likeness. LogP values between −0.5 and 5 reflect balanced lipophilicity and absorption potential. Total polar surface area (TPSA) values under 140 Å^2^ suggest good oral bioavailability. The number of hydrogen bond donors (HBD ≤ 5) and acceptors (HBA ≤ 10) complies with Lipinski’s rule, indicating favorable pharmacokinetic properties.

**Table 7:** ADMET properties of the lead ligands and geldanamycin.

Category	Property (Unit)	Lig 1	Lig 2	Lig 3	Geldanamycin
Absorption	Caco-2 Permeability (cm/s)	−6.426	−5.256	−4.767	−5.246
	HIA	>>30%	>>30%	>>30%	<30%
Distribution	BBB Penetration (cm/s)	0.0	0.039	0.179	0.0
	PPB (%)	64.831	92.445	95.06	86.92
Metabolism	CYP1A2 inhibitor	0.0	0.0	0.0	0.0
	CYP2C19 inhibitor	0.0	0.0	0.006	0.0
	CYP2C9 inhibitor	0.0	0.029	0.174	0.0
	CYP2D6 inhibitor	0.0	0.0	0.0	0.0
	CYP3A4 inhibitor	0.0	0.003	0.121	0.0
Excretion	CL (mL/min/Kg)	3.34	12.456	12.709	8.675
	T½ (H)	2.758	2.065	1.625	0.881
Toxicity	hERG Blockers	0.025	0.046	0.057	0.024
	AMES Toxicity	0.386	0.665	0.81	0.702
	Skin Sensitization	0.986	0.965	0.988	0.973
	Respiratory Toxicity	0.109	0.835	0.912	0.046

Note: High human intestinal absorption (HIA > 30%) and strong Caco-2 permeability indicate good oral absorption. Low blood–brain barrier (BBB) penetration (<0.2) reduces the risk of central nervous system side effects. Minimal inhibition of CYP450 isoenzymes suggests low potential for drug–drug interactions. Clearance (CL) and half-life (T½) values reflect the compounds’ metabolic stability, while negative hERG and Ames test results indicate low cardiotoxicity and non-mutagenicity.

**Table 8:** Prediction of the toxicity of the lead ligands and Geldanamycin using the Protox-3.0 platform.

Ligands	Lig 1	Lig 2	Lig 3	Geldanamycin
Hepatotoxicity	Inactive	Inactive	Inactive	Inactive
Carcinogenicity	Inactive	Inactive	Inactive	Inactive
Mutagenicity	Inactive	Inactive	Inactive	Inactive
Cytotoxicity	Inactive	Inactive	Inactive	Active

Note: Negative Ames test results indicate non-mutagenic potential, while non-carcinogenic and non-hepatotoxic predictions support a favorable safety profile. The absence of cytotoxic activity further suggests that the compounds are safe candidates for drug development compared to the reference inhibitor.

## Data Availability

Data are contained within the article. Further inquiries can be directed to the corresponding authors.

## Supplementary Materials

The following supporting information can be downloaded at: https://www.mdpi.com/article/10.3390/cimb47100860/s1.

## Abbreviations

The following abbreviations are used in this manuscript:

ADMET	Absorption, Distribution, Metabolism, Excretion, and Toxicity
BBB	Blood–Brain Barrier
BP	Biological Process
CC	Cellular Component
CYP	Cytochrome P450
HIA	Human Intestinal Absorption
HSP90	Heat Shock Protein 90
IC50	Half Maximal Inhibitory Concentration
MD	Molecular Dynamics
MW	Molecular Weight
NF-κB	Nuclear Factor Kappa-light-chain-enhancer of Activated B cells
PDB	Protein Data Bank
PPI	Protein–Protein Interaction
PPB	Plasma Protein Binding
RMSD	Root Mean Square Deviation
RMSF	Root Mean Square Fluctuation
SA	Synthetic Accessibility
SP	Standard Precision (Docking mode in Schrödinger Glide)
TPSA	Total Polar Surface Area
