# Supplementary material for: In Silico Network Pharmacology, Molecular Docking, and Molecular Dynamics Analysis of Rosemary-Derived Compounds as Potential HSP90 Inhibitors for Cancer Therapy
Source: Curr Issues Mol Biol. 2025 Oct 18;47(10):860. doi: 10.3390/cimb47100860 (PMC12562389; doi:10.3390/cimb47100860)
Supplement: Supplementary file 1 [file cimb-47-00860-s001.zip › cimb-3911231-supplementary.pdf]

In Silico Network Pharmacology, Molecular Docking, and Molecular Dynamics Analysis of Rosemary-Derived Compounds as Potential HSP90 Inhibitors for Cancer Therapy

Radhia Mazri[1], Mebarka Ouassaf[1], Afaf Zekri [1], Kannan R. R. Rengasamy[2,3] · Shaf Ullah Khan[4,5] · Bader Y. Alhatlani[6]

Table S1 Results of Shared and Unique Gene Analysis Based on Venn Diagram

Names	total	elements
Antitumor Rosemary	178	P03956 P19793 P23975 P18054 O60218 P04150 P17948 P53779 Q9UBN7 P07949 P35354 P11362 P16581 P04629 P51580 P14679 P09917 P09874 P42226 P10826 P14780 P04637 P10415 P48147 P11413 P06493 P42574 P10275 P03372 P08185 P01375 P17516 P00533 P35372 P10828 O75874 P25025 P08263 P07947 P04626 Q8TDU6 P11712 Q9GZV3 P11511 P12931 Q04609 Q9NR96 B2RXH2 P42330 Q99808 P62942 P08069 P09619 Q99683 P00734 P49841 P05129 P13866 Q9HC97 P45983 Q92731 Q16790 Q15465 Q16678 P21728 P55210 P55055 O75164 P07339 Q9UF33 Q9H4B7 Q14534 P23219 P08246 P40763 P04818 P20839 P14174 P48736 Q13547 Q13627 P16083 P24941 P08183 Q09472 P35228 P04798 O00767 P37840 Q05932 P11387 P34972 P08581 P08473 P30307 O14746 O14920 P07948 P06276 P53671 P11309 O75469 P28482 P17936 Q12884 Q05513 P15144 P09237 P16050 O60706 Q16236 P36507 P12268 O00206 P50416 Q16539 P35968 P00492 P17252 P30305 P42575 Q03181 P29466 P53350 P36888 P13631 P07858 P30304 P18405 P11802 Q9NYA1 Q9UNQ0 P08253 P11473 P15559 Q05655 P30968 P30518 Q16665 Q8NER1 P08684 P50281 P42338 P43405 Q14790 P06401 P06746 Q9UM73 P56817 Q04206 P05177 P60568 P15121 P04278 P25101 P24723 P08254 Q96RI1 P42345 P27361 P05067 P11388 Q04759 P42336 P45452 P04062 Q14289 Q02156 P13569 P33261 P06213 P10276 P13726 P24666 P08842 Q06187 P07900 Q00987
Rosemary	328	Q13882 Q13946 P31213 P29371 P08173 P21462 P12821 O43570 O60674 P18825 P46663 P34995 P60033 P24864 P24941 Q86YT5 P31645 P55201 P29320 P21397 P32239 P56373 P41145 P20309 P35348 P48039 Q13093 P37059 P42681 P32245 Q9NYQ3 P43235 Q9Y4C1 Q92753 P23280 P06239 Q15746 P11474 Q00535 Q01959 P00797 Q16850 P11217 Q9Y2J8 P16109 Q06124 Q9Y463 O75116 Q13258 Q07869 P54756 O15379 P43116 P20701 P05362 P05107 P52732 P09769 Q16548 P14416 P08138 P05186 Q9Y5Z0 Q9UM07 P05556 P13612 P40189 P55212 Q16584 P22894 P51449 Q15858 P51531 P55072 P02753 Q08881 Q01433 P29350 P15086 P35398 P00746 Q9ULX7 Q9ULC6 Q2M385 Q14524 P00918 Q14416 P13497 P30556 P23786 O75173 P28845 P47712 P17655 P08912 Q9NZJ5 P11229 P30559 P80192 P35218 Q01064 O76083 Q8IXJ6 Q92843 P21554 P0DMS8 P30531 Q12772 P50052 P29317 O95718 P04066 O14786 P18089 P31639 Q14994 P35916 Q14330 P43166 P15090 P48443 O75908 P54577 Q9NRA0 P08172 Q07343 P14151 P30542 P28838 Q13003 P07711 Q8WWL7 P06493 P14635 O95067 P28472 P18507 P14867 P23458 P00338 P09960 P10586 Q13464 P24530 P51813 P51451 P04035 P29275 O96020 P24941 P24864 O60885 P17706 P14927 Q96P20 P41240 P00915 P08235 Q9Y256 P08311 Q9UQB9 P32247 P33316 Q07075 O00408 P30536 P10619 Q92523 P29323 Q9Y2R2 P51686 Q9Y2T6 P32238 Q9NUW8 P05093 O43293 P07451 P98073 Q9UNI1 Q96D53 P04746 Q9UNA0 Q9H999 P37058 P49286 Q92769 P24385 P11802 Q15349 P02766 P15085 P53609 P49354 P51512 P18031 P22748 P19021 Q9GZT9 P05091 P09467 P19099 P54764 P49354 P49356 P14867 P28472 P18507 P31644 Q9H3R0 P56545 P52333 P21731 O15055 P41146 Q02127 Q9NPH5 P06737 Q16602 Q01432 P51681 Q8N1Q1 Q8WWR8 O43353 Q15761 Q12791 P26010 P13612 Q99527 O00519 Q8TDS4 P28472 P34903 P18507 P23141 O76074 Q08499 P41594 P16662 Q9UBS5 P20292 O43613 P24593 P47869 P28472 P18507 P09884 O14684 Q9H2K2 Q9Y3R4 P25100 O60911 P35368 Q9UQ49

		O75460 Q13443 O15229 Q9Y3Q0 Q06418 P54753 P32246 P14061 O43614 P04054 Q13133 Q16875 P49759 O60341 O43826 Q01469 P31939 P35610 P34998 P23946 Q6P179 O60427 Q9UBU7 O00311 P34913 Q96RR4 P80365 P48449 P22303 P08913 Q7Z2W7 P15538 P08709 Q9BY41 Q9Y2D0 P52895 P36639 P27338 P05413 P23470 P49810 Q9NZ42 Q92542 Q96BI3 P49768 Q8WW43 P11940 P07477 P29274 O14842 P06241 P37231 P24941 P78396 P20248 P15382 P51787 Q86V86 Q99685 Q9P1W9 Q96PN6 P53396 P10827 P42892 Q99572 Q9UHC9 P07384 P04632 Q8NG68 Q07912 P07148 Q9BYT3 P50406 P39900 P00742 Q02779 P07384 P53609 P22460 P39086 Q9Y5Y4 Q8WUI4 Q13002 O75688 Q05193 O00748 Q9Y233 Q15078 Q00535 P36873 P50579 P49238 O95271 P48058 P28702 P11766 Q9ULW8 O75762 P41595 Q5S007 Q99720 P21917 P07333 P23109 P41143 P29597 P21709
Antitumor	913	Q9UBE0 Q5R3K3 P10323 Q96EY1 P01215 P30101 Q13489 P30872 O15244 P40198 P33260 P23258 O00443 P01574 P27986 Q99816 P11926 P21980 Q6NVY1 P04198 Q99933 P0DN86 P20585 P05305 Q15759 O15440 P25942 P19113 P01008 P49327 P02751 P46013 P35498 P08123 P14778 P20809 P10645 P18848 P20366 Q07812 P21359 Q00613 Q7L0J3 P00451 P26045 Q6L8Q7 P09104 P00441 Q99958 P25874 P51587 O75306 Q9UBN6 Q8N122 P35638 O15455 P15514 P01135 O94788 P35790 O14757 P13501 P20815 Q07864 Q9HD33 O00300 P0DSE2 Q9Y253 O00329 Q9UPY3 O94806 P53985 O15519 P40225 P10253 Q06830 Q13233 P43351 Q13126 Q15399 Q14764 O75715 P29353 P55036 P29401 P04141 P62979 O00555 Q92831 Q92889 P13498 P14672 P16410 P01911 Q16206 Q01973 O15431 P27694 P98160 P11168 Q13077 Q05086 Q13586 Q14145 Q9UHN1 P04271 O15539 Q02750 P01563 Q13114 P11836 P01241 P01730 P27816 O60896 P15428 Q13546 P32929 Q9BXW9 P31749 P16035 P30530 P21589 Q9Y2I7 P28074 P38432 Q14683 P20813 Q13490 Q99700 P12956 P07476 P10071 P48775 Q99549 Q01831 P15976 Q9NWW6 O94768 Q9BZL6 P27707 Q9NYJ7 Q8N118 Q96FX8 Q8WYR1 P05112 P69891 Q12923 P11172 Q9P104 P12314 Q6P589 O15264 P04155 Q9Y5Y9 P25705 P15328 Q9Y2C9 O60733 O00303 O96017 O14727 Q8WUF5 Q15517 P11021 P01130 P55000 Q13485 Q9NP85 Q6NUS8 P10809 P13693 P45985 P0DMM9 P43630 Q13936 P13500 P10721 P38117 Q6IA69 Q9UBC7 P22732 Q05397 P05783 P43234 Q92830 P13612 P19174 P35789 P40306 P21912 O14618 P43246 P49959 P07101 O14763 Q6P597 Q16621 P35070 P33527 P35503 P01579 Q13526 P08833 P16152 P56693 Q14738 P16860 Q07325 Q8WTR2 P83111 P02749 P04792 Q8IWU5 Q9UIG5 P05181 P60510 P61278 P46531 P10145 Q9NYW4 P51959 Q9H492 Q00975 P15018 Q12809 P07992 P50591 Q06710 P53667 P09488 P35900 P16949 P49023 P28065 Q9Y2D1 Q8N2Q7 P22607 P07996 O95342 P08700 P12004 Q07817 P00450 Q96QE3 P01588 P20674 P22004 Q9NZQ7 Q53H12 P06454 P11586 O75594 P49916 Q8WWT9 Q15080 Q16082 P40933 P23468 P04731 P35670 Q13885 P05231 P15151 Q14152 P35225 Q9UL12 Q08722 P01034 P01100 Q00653 P00387 P06731 P16104 P04040 Q6PEY2 Q9NPI8 P63261 O43524 Q96CA5 Q07065 P17676 P16389 Q11206 P06881 P05787 Q8NBP7 P35658 P67870 P00966 P13232 Q8IZR5 Q9H1B5 P30711 Q08945 P00519 P11465 P06400 O60603 P34896 Q8WXH0 Q16513 P09038 O60502 P35557 P40126 Q9UJT0 Q969H0 P68400 P40313 P21964 Q9UIF7 P24864 Q14159 O95985 P18075 Q96EB6 Q9UBR1 P19438 P29474 P05113 Q9NRR4 P19838 Q9NW38 Q9UEY8 P41743 Q8IVT2 Q00597 P50226 P01106 Q6GPH4 P05164 P08637 P23921 Q9BYW2 P31350 Q6QHF9 O14578 Q9H3D4 Q99728 Q9C035 Q13393 Q92569 P43694 Q6NYC8 Q13535 P16455 P27169 P08670 P01137 Q9H1J7 P27695 P20591 Q9Y6K9 P07585 P21266 P06748 P52926 Q86XF0 P30626 P28300 P00740 P11245 P07988 P51608 P24462 P16070 O43405 Q96JB1 P23771 Q9Y6Z7 Q13158 Q9NY61 P04350 P05771 P04053 P68363 P30411 P56975 P05121 P07204 P04070 P0DPH7 P11226 P02787 P54652 P10909 P49815 P01111 Q9UKT9 P22079 Q02297 P10321 O00459 P00747 P11274 P14136 Q8N726 P43403 Q9UER7 Q99558 O15392 Q12981 Q92887 Q13426 P15056 P01583 P30279 P55957 P15941 P02686 Q99707 P20783 Q14624 O15439 Q9NYK1 P49771 Q01196 Q9P2G4 P79483 P01222 P00709 P38936 Q15393 P28907 Q02880 P02795 Q15139 Q13459 Q99714 O75881 P39748 P19971 P35240 Q14191 P15692 Q96PZ7 O43542 Q9NPF4 Q14978 Q15796 Q14244 Q3MIW9 P39687 P14555 D6RGH6 O75015 P09601 P22301 P04196 P11509 P32970 Q07108 P29460 P62875 P02778 P47710 P48634 P05107 P00167 Q13867 Q9Y243 O75897 Q14117 P61073 P37287 P12643 P02741 Q16831 O00255 Q96KQ7 Q07666 P18509 O75496 P10599 Q9NR28 Q96LT9 Q9P1U0 O00750 P38935 Q99570 Q8NEB9 P09429 O14497 Q8NG50 P18887 Q9NRZ9 P22352 Q16620 P54132 P15336 P40121 P60484 O14980 Q12778 P16435 P46100 P11142 Q6N021 P28906 Q3ZCM7 P14902 P14635 P02771 P04179 O75251 Q14654 Q9UKV0 Q8TBQ9 O95980 Q93052 Q9HCN6 P12318 O60669 P48506 Q9UBT2 P35869 P22314 P12955 P14210 O95905 P40337 P07288 Q03164 P05543 O15432 P25105 P51570 Q8NEV9 P99999 Q5FYA8 P15036 P12259 P08922 P17693 O75807 Q9H853 O00233 O60573 Q04656 P10147 P21802 O00625 Q16822 Q71U36 P12272 O43488 P14410 P62826 P08151 Q07820 P22309 Q5SQI0 Q9HB55 P01116 Q8WXI7 Q13043 O15287 P17735 P00374 P00326 Q92748 Q9Y6M1 Q9NST1 Q15070 P01138 Q02817 Q01094 P51797 P19634 Q9BUF5 P05412 P21583 P12035 O00423 Q9NPH3 O95163 O75751 P61769 Q9P2P1 Q14344 Q9HAW7 P00813 P35232 P61981 P25786 P02144 P51589 Q9Y694 P37275 P52948 P25963 P24855 P62877 P54278 P07437 P26358 O14617 Q495A1 P24522 O76064 P30044 Q969S8 P21817 Q9Y230 Q9NY65 P19235 P21781 P35408 O43196 P67809 P51787 Q9NWM0 Q86TM3 P08631 Q8N8D7 P84022 Q13315 Q6P5Z2 O15297 P19256 P28562 P06127 P11137 P17661 O94907 Q92521 P36542 Q9UKK6 Q9NRH3 P10632 O75489 P41235 P34932 Q9Y6D9 Q69384 Q9BW66 Q9P209 P02768 P19440 Q15257 P26447 Q02246 P47989 P27708 P15260 Q13131 Q03135 P00156

		Q9BXI3 P29372 O60469 P53778 P12830 O95255 P52701 P07307 P05814 O60673 P07492 O43281 P42858 P60709 Q9BTV4 Q92934 Q9NX76 Q9HCN2 Q02447 Q13257 P01854 P46059 Q9UNA4 P09564 P05187 Q86UG4 Q76LX8 P17405 Q9UM21 P40692 P24385 P68366 P10635 Q6ZQN7 P51654 P01562 P20273 Q9NZR2 P04114 O94956 P11166 P52951 P06576 P46063 P09211 P31751 P08758 P41182 Q6UVK1 P42898 Q5UE93 Q13427 Q9UNE7 Q9UQ84 Q9NYV4 P62745 P15172 P08047 P02818 Q8TC92 Q9BQE3 P31944 Q9UIF8 O15360 Q14213 P35475 P40305 Q15389 O00220 O75828 P15153 P38398 P08238 Q16739 O15111 Q8WUG5 P07203 Q92793 Q9UIQ6 P04049 P0DMV8 P98170 Q16512 P80294 P19544 O14788 P61812 P25445 P41440 P0DPH8 Q9BVA1 P18074 P43487 P14550 O14798 Q5T3U5 Q96FL9 O15245 Q96MW7 Q8NF50 P19224 P27487 P49356 Q8TEW8 P29475 Q12882 P01133 O15350 Q96IZ0 P14784 P01589 Q00534 P59534 P02786 P49888 P45844 Q14116 O94782 P04180 P84996 P01112 O95865 Q9BVP2 Q9NR19 P83916 P17931 P20248 Q15672 Q13740 O95831 O60656 Q15717 P28062 Q06609 Q8WWK9 Q14008 Q8TDM6 P07099 Q9Y6K1 P45984 Q13309 Q9NPD5 P23025 P01033 Q9NQS5 P21673 Q9NP60 P55211 P48061 P23919 Q7L5Y1 P19320 O95045 P21860 Q9UBT6 P35222 Q9HB96 P30838 Q16611 P21399 Q9NR97 P13591 P10242 Q8NGJ1 P08887 B1AK53 O15438 P49792 O15427 Q86VW1 Q9Y6L6 O75330 Q14397 P00749 P05154 Q9NNW7 P46527 P16671 P09210 Q9NXP7 P10747 P43155 P35568 P29965 P68871 P20701 Q04828 Q9NS18 Q06203 P10636 O15530 Q13509 P33681 O95477 P00738 Q01974 Q96J66 P15529 P08236 P08397 Q14956 P30281 Q8TBE0 P50225 P08575 P52757 Q12933 Q06546 P17813 P13686 O75473 O14965 P23769 P78380 Q15311 O60566 O43543 Q8NET8 O75909 P42166 P08865 Q04760 P09919 P22455 P54802 Q9NVD3 Q07973 P02788 P20810 P00390 P49189 P01189 P42224 Q7LG56 Q15327 Q9Y478 O43181 P17302 P00352 P04406 P23560 A6BM72 Q6UXA7 Q9HAS3 P09603 Q15942 O15446 P18146 O75747 O60934 P21439 P01584 P68371 Q07866 Q15831 P48023 Q9UJT1 P15391 P04275 P21675 O15296 Q9NUT2 P13010 Q92574

Table S2. Different parameter scores of Enrichment

ROC	BEDROC (α=160.9)	BEDROC (α=20)	BEDROC (α=8.0)	RIE	AUC
**0.93**	0.657	0.794	0.845	8.10	0.89

Table S3: Count and percentage of actives in top N% of decoy results.

%Decoys	1%	2%	5%	10%	20%
**#Actives**	23	29	30	30	32
**%Actives**	65.7	82.9	85.7	85.7	91.4

Table S4: Count and percentage of actives in top N% of results.

%Results	1%	2%	5%	10%	20%
**#Actives**	6	13	29	30	32

**%Actives**	17.1	37.1	82.9	85.7	91.4

Table S5: Enrichment Factors with respect to N% actives recovered.

**%Actives**	**40%**	**50%**	**60%**	**70%**	**80%**	**90%**	**100%**
**EF**	20	21	21	20	20	4.7	1.1
**EF***	57	73	75	69	62	5.4	1.1
**EF'**	53	55	57	58	59	11	2.2
**FOD**	0.004	0.005	0.005	0.005	0.007	0.02	0.07
